# The potential role of network-oriented interventions for survivors of sexual and gender-based violence among asylum seekers in Belgium

**DOI:** 10.1186/s12889-020-10049-0

**Published:** 2021-01-05

**Authors:** Emilomo Ogbe, Alaa Jbour, Ladan Rahbari, Maya Unnithan, Olivier Degomme

**Affiliations:** 1 International Centre for Reproductive Health, Ghent University, Corneel Heymanslaan 10, 9000 Ghent, Belgium; 2 Centre for Research on Culture and Gender, Ghent University, Blandijnberg 2, Ghent, 9000 Belgium; 3 Department of Social Anthropology, Sussex Centre for Migration Research, International Development, University of Sussex, BN1 9RH Brighton and Hove, United Kingdom

**Keywords:** Social support, Asylum seekers, Violence, Network theory, Sexual and gender-based violence

## Abstract

**Background:**

Social support and social network members have been identified as an important factor in mitigating the effects of sexual and gender-based violence (SGBV) and improving the coping process for many survivors. Network oriented strategies have been advocated for among domestic violence survivors, as they help build on improving social support and addressing factors that alleviate repeat victimization. There are opportunities to implement such strategies among asylum seekers who are survivors of SGBV in asylum centres, however, this has not been fully explored. This study sought to identify key strategies and opportunities for developing peer-led and network-oriented strategies for mitigating the effects of SGBV among asylum seekers at these centres.

**Methods:**

Twenty-seven interviews, were conducted with service providers (*n* = 14) / asylum seekers (*n* = 13) at three asylum centres in Belgium. A theoretical model developed by the research team from a literature review and discussions with experts and stakeholders, was used as a theoretical framework to analyse the data. An abduction approach with qualitative content analysis was used by the two researchers to analyse the data. Data triangulation was done with findings from observations at these centres over a period of a year.

**Results:**

Many of the asylum seekers presented with PTSD or psychosomatic symptoms, because of different forms of SGBV, including intimate partner violence, or other trauma experienced during migration. Peer and family support were very influential in mitigating the effects and social costs of violence among the asylum seekers by providing emotional and material support. Social assistants were viewed as an information resource that was essential for most of the asylum seekers. Peer-peer support was identified as a potential tool for mitigating the effects of SGBV.

**Conclusion:**

Interventions involving asylum seekers and members of their network (especially peers), have the potential for improving physical and mental health outcomes of asylum seekers who are SGBV survivors.

**Supplementary Information:**

The online version contains supplementary material available at 10.1186/s12889-020-10049-0.

## Background

Social support and the positive influence of social network members are important factors in mitigating the effects of sexual and gender based violence other forms of violence and life stressors, as well as improving the coping process for many survivors [1, 2].

In this article, we refer to social support as ‘comprising both the social structure of an individual’s life and the specific functions served by various interpersonal relationships’ [3] We also define sexual and gender based violence (SGBV) as any act that is perpetrated against a person’s will and is based on gender norms and unequal power relationships [4]. Sexual and gender-based violence also encompasses threats of violence and coercion. It can be physical, emotional, psychological, or sexual in nature, and can take the form of a denial of resources or access to services. It inflicts harm on women, girls, men, and boys. There are currently knowledge gaps on the processes through which peer-support mitigates consequences of sexual and gender-based violence, among asylum seekers, in existing peer-support interventions.

Many asylum seekers experience sexual and gender based violence during their migratory journey from the countries to Europe, as well as in the destination countries [5], men, women and young children are all victims of SGBV but women and children are the most vulnerable [6]. The consequences of sexual and gender-based violence are limited not only to physical consequences, but also psychological effects as well, like injuries, gynaecological disorders and mental health disorders, most commonly post-traumatic stress disorder [7–9]. In this paper, we define PTSD within this study as ‘a mental health condition that’s triggered by a terrifying event — either experiencing it or witnessing it. Symptoms may include flashbacks, nightmares and severe anxiety, as well as uncontrollable thoughts about the event’ [10]. For asylum seekers, the added layer of vulnerability due to their experience of escaping a humanitarian setting and stressful experiences of migration, makes disclosure and help seeking difficult, especially considering they are in a different context without their regular sources of social support and assistance [6].

The mechanisms and processes through which social support affects coping processes is complex and time dependent. It is also reliant on the structure of the social network and inherent capabilities of the individual [11]. A study comparing the social networks of women in abusive relationships with their domestic partners, compared to ‘non-abused’ women, found their social networks to be smaller, with fewer reciprocated ties. These women were also more likely to provide support than receive it, compared to non-abused women of the same socio-economic group [12]. Although abused women often played a ‘central’ role in their networks, mostly serving as a link between different members of their network and as a resource person, they had few people they discussed their problems with.

Survivors of SGBV will seek help first through informal sources (friends and families) before more formal sources like medical centres and legal assistance [13]. Several reasons are often cited for this, some of these include sociocultural beliefs around sexual and gender-based violence, and the stigma and shame associated with seeking help from formal sources. Other reasons cited is the feeling that these formal sources (judicial, health centres and shelters) might not provide the required support needed. In these cases, survivors of violence were more likely to discuss their experiences of violence with close friends and family [14, 15]. This, elucidates the need for closer attention to network members of survivors of violence when developing interventions [2]. This is especially true for asylum seekers, refugees and undocumented migrants, with the same national and ethnic identity, among whom stronger ties might exist than with service providers or members of the destination country [16]. Smith’s work on female refugee social networks revealed an evolving social network structure, with strong homogenous ties among people with similar national identities, and weak social ties with people from the host country. Weak ties refer to social network members that do not have a strong influence, live far from the survivor of violence, are not part of their everyday lives, especially relationships that are not reciprocal and the asylum seeker does not consider high value. The importance of this statement is related to asylum seekers, refugees and Internally displaced persons who due to migration or displacement have been separated from family and friends with strong social ties and now live-in countries where they have weaker social ties/ connections with people around them.

Network-oriented strategies have been advocated for among SGBV survivors, as they help build on improving social support and addressing factors that alleviate repeat victimization [17]. They have also been used among other vulnerable populations like intravenous drug users [18], for HIV risk reduction strategies [19] and to provide support for individuals with chronic conditions like diabetes [20]. There are opportunities to implement network-oriented strategies among asylum seekers, for example, involving family members and peers in mental health interventions, group therapy sessions [21] for interventions similar to mentor mothers [22]. There are already existing network oriented interventions, specifically family-oriented interventions, focused on the mental health of asylum seekers and refugees’ which have been found to be successful [23]. These strategies could also be implemented in different humanitarian settings. In this study, we have defined asylum seekers as ‘someone whose request for sanctuary has yet to be processed by the host country [24] .

This study sought to understand the social network and support characteristics of asylum seekers and refugees at three different asylum centres in Belgium. Consultations with different stakeholders and findings from the interview were used to develop a theoretical model that explains the motivating factors and thought processes involved in decision making and factors associated with their social network that affect disclosure of intimate partner violence survivors. The aim was to identify key strategies and opportunities for developing peer-led and network-oriented strategies for mitigating the effects of sexual and gender-based violence among asylum seekers at these centres. In this paper, network interventions are defined as ‘purposeful efforts to use social networks or social network data to generate social influence, accelerate behaviour change, improve performance and/ achieve desirable outcomes among individuals, communities, organizations or populations [25]. We also refer to ‘peer-led’ interventions or ‘peer to peer’ support as interventions led by, or support from other asylum seekers, who have experienced sexual and gender-based violence. This could be in the form of mentoring and providing information and referrals, or through online or group forums [26]. As a result of consultations with experts and findings from interviews with asylum seekers and service providers, we developed a theoretical model to explain the ‘pathways’ and factors that determine how actions of network members influence the decision to access health care services and the different outcomes of these processes [27].

### Theoretical model

The development of the theoretical model was based on:

i) a literature review of network and social support theories and interventions among survivors of sexual and gender-based violence
ii) In-depth discussions with asylum seekers who were survivors of violence, experts in the field of migrant health, intimate partner violence and other forms of sexual and gender-based violence, as well as economic theorists with expertise on network effects and game theory.

**Fig. 1:**
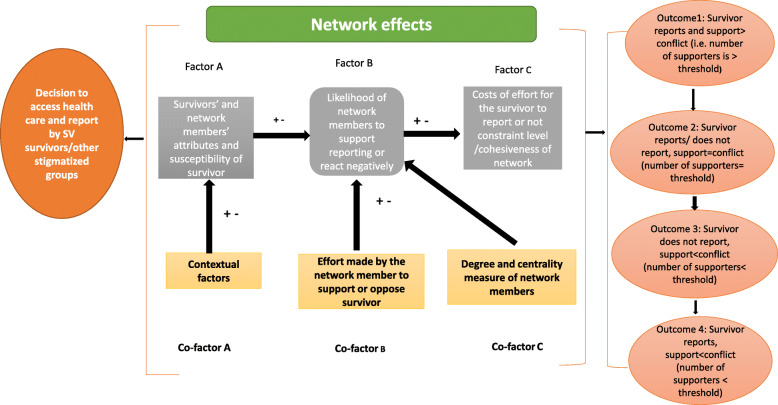
Model explaining network effects on reporting behaviour of intimate partner violence survivors

The added value of this approach was to ensure that the model reflected the realities of support structures of many asylum seekers, refugees, and sexual based violence survivors, as well as the important factors that influenced their decision making. The model and influencing factors is depicted in Fig. 1 [27].

The proposed model discusses the way actions and efforts made by network members can positively or negatively influence decisions to access sexual and gender-based violence. We have explained the different factors of the model. The underlying assumption of this theoretical model is that different factors influence decision making regarding reporting an incident of sexual and gender-based violence, at the interpersonal level, within a survivor’s social network. For example, a survivor, who has more access to resources, is literate and is situated within an asylum centre, with SGBV reporting policies, screening and referral availability, is more likely to have members of their social network within the asylum centre, who will support reporting the incident of SGBV. The effort made by the social network member to encourage the survivor of SGBV to report or not report, will have a large influence on the decision as well, however, this is highly determined by the power and influence, this network member has. So, the level of influence of a close friend or relative is determined not only by the existence of the relationship but by the quality of the relationship, and how well connected the network member is.

The network factors outlined in the theoretical model are discussed below:

### Factor A. survivors and network members’ attributes

These refer to ‘intrinsic’ characteristics of the survivor and network members, for example, gender, age, ethnicity/ race, and other related characteristics that could be defined as sociodemographic. An additional factor, we included into this category, is the concept of ‘resilience’- which we define as the ability of asylum seekers who are survivors of sexual and gender based violence to cope with the psychological consequences of their experiences and trauma during migration, and navigate the challenges of adapting to a host country in a hopeful yet pragmatic way [28].

### Contributing co-factor, a: contextual effects

These are ‘extrinsic’ attributes for example the refugee camp or centre, existing laws and regulations regarding access to SRH services for asylum seekers, availability of infrastructure and sufficient staff to address these issues, as well as a reporting mechanism for reporting cases of gender-based violence.

### Factor B. network members’ reaction to the survivor reporting

This refers to the probability or chance that network members will react positively or negatively to the decision of the refugee to seek out health care services.

### Contributing co-factors B

i) Effort required by the network members to support or oppose: We assume that the network members of a survivor of SGBV are rational in thought. Hence, if it takes too much effort to support the survivor to seek healthcare services, this will influence their decision to provide support. Hence, the higher the personal effort or cost to support the survivor of violence, the more likely they will be neutral or oppose the decision of the survivor to report or seek healthcare. An example of effort could be the financial cost, time cost or emotional burden of providing support.
ii) Degree and centrality measures of network members: Degree and centrality measures refer to the ‘power’ or level of influence, the network member has within the refugee’s social network. High degree (highly influential and connected) members who oppose or support the refugee to access health care service, will have more influence on the decision making and the actions of other network members, than network members with few strong ties and lower levels of influence.

### Factor C: constraint and cohesiveness of the network

In our model, we assume that within a network, the extent to which, network members’ actions and perceptions can prevent the refugee from reporting or accessing health care is dependent on how cohesive the network is; by ‘cohesiveness’ we refer to the strong ties between groups, that ensure the members of the group remain linked.; A constrained ego (network member) within a cohesive network, is one in which the other people in the network are connected to each other, and the ego’s actions and perceptions are controlled by his or her personal network [25].

### Threshold and resilience

In our model, we propose that the additive and detrimental effect of these factors will result in several outcome scenarios based on the idea of a threshold. Survivors of violence will seek health care with positive consequences if the additive effects of Factors A, B and C and their co-factors exceed this required threshold level. Below this threshold level the benefits of reporting would be non-existent, or reporting might cause the refugee such negative consequences, that it is not in their interest to seek health care services.

In developing this concept, we also take into account unexplained characteristics like resilience, which we understand is difficult to measure. When we define resilience, we refer to the innate ability of a survivor of sexual and gender-based violence to cope with external stressors and challenges, in spite of the absence of resources and support. By mapping out these factors, we hope to provide a way to map out with qualitative and quantitative factors, the way social networks affect decisions to access health care among asylum seekers, not only in cases of sexual and gender-based violence but other stigmatizing situations, and for example when refugees are dealing with mental health issues or infectious diseases like HIV/AIDS.

### Outcomes from network effects

We describe several potential outcomes based on the concept of a ‘threshold effect’, looking at the summation of ‘positive’ and negative influences of network members, as well as intrinsic and extrinsic factors that might influence decision making, already explained above. We have divided these outcomes based on whether the level of support is equal to, less than or more than the level of conflict and the decision the survivor takes.

- Outcome 1:Survivor reports, and the level of support is greater than conflict, which would be the best outcome for the survivor. In this scenario, the survivor will require less effort to report (hence, less individual cost), as their network members will provide sufficient support and resources (information, emotional and monetary support) to mitigate whatever stigma or negative consequences they will experience, after reporting.
- Outcome 2: The level of support and conflict is equal, but the decision to report is based on survivors’ perceived benefit of reporting, as opposed to the actual benefit. In this scenario, there are several challenges to reporting their experience of sexual and gender-based violence and seeking health assistance. These challenges are equal to whatever support or benefit the survivor might gain from seeking care. Hence, the decision to report is more heavily influenced by the ‘perceived’ personal cost to the survivor of violence, and less on the existing challenges or benefits. We assume that most people in this situation, do not report or seek health care, unless there is an intervention.
- Outcome 3: The level of support is less than the level of conflict caused by reporting, but the survivor decides to report. In this scenario, the consequences of reporting, for example stigma, loss of resources and support network, etc., far outweigh the ‘social’ benefits of reporting. However, the survivor goes ahead and reports their experience of violence. In this situation, this survivor of violence requires more support from health workers, as well as psychosocial counselling and follow up. These survivors might be viewed as having more resilience, but are actually in a more vulnerable situation, as a result of seeking care.
- Outcome 4: The level of support is less than the level of conflict caused by reporting, but the survivor decides to not report, in this scenario, the survivor of violence makes a rational decision to not seek health care because of the negative consequences of reporting. However, in so doing they are unable to get treatment and the needed psychosocial counselling required. Also, in an instance where the perpetrator(s) are part of the family, there might be repeat incidents of abuse.

## Methods

Different qualitative research methods were triangulated to cross-validate research findings. A combination of ethnographic methods, specifically observations were combined with key in-depth interviews between November 2016 to February 2018. The total number of hours spent on observations, was 862 h. Interview guide questions and themes are attached to this manuscript as an appendix. The observations were conducted in three centres, two of these centres were located in East Flanders and the third was located in Brussels. See Table 1 which provides more detail about the centres and time allocated to each centre. See Table 2 which provides specific information about service provision at the centres. Ethnographic methods were employed to help us understand the pathways of care and the daily life experiences of people who lived in the different centres selected for the research. Observations involved following-up with consultations, assisting with daily tasks required in the centres’ and attending social events with the refugees. Selection of the centres was purposive and done in collaboration with the Director of Medical services for the Federal Agency for the Reception of Asylum seekers (Fedasil), as well as with researchers and service providers working with asylum seekers. The purpose of choosing different types of centres was to assess how the structure and organization of asylum centres and policies influenced the perception of support by the refugees in the centres and the relationship between the service providers and the asylum seekers. Ethics approval for the study was gotten from the Committee for Medical Ethics, University of Ghent teaching Hospital. Approval to conduct the study at the asylum centres, was obtained from FEDASIL. Purposive sampling was done to ensure that research participants were a mixed group of service providers (social workers (2), psychologist (1), nurses (3), education workers (2), medical doctors (4) and asylum seekers (13) (men, women). Informed consent was obtained from all participants and permission taken for audio recording. See Table 3 for summary of research participants” information.

**Table 1:** Summary of information about centres and observation activities at study sites

Name of centre/Location	Description of centre	Type of centre	Activities done/observed	Length of observation	General description
Centre 1	Located by the port, in an old ship. Capacity: 250 people. Majority of the residents were of Syrian, Iraqi, Iranian and Afghan origin. A combination of families and single persons. Rooms were for 4 to 6 people in bunks. No specific demarcation of male and female spaces	Open-access centre	Consultations with the doctors, nurses, social workers, and education workers. Provided support for clinic consultations and spoke with and interviewed refugees at the centre	6 weeks/ 6–9 h per week: November to December 2016 (Centre closed February 2017)	Medical and social services were located within the centre. However specific opening hours were allocated for service provision. The nurses and education workers were more accessible to the refugees, the doctors much less so. Free entry into the centre by refugees (i.e., entrance not manned by security officers or no barriers or gates), however they were allowed only specific days to live outside the centre. Private consultation rooms were available that allowed for a certain level of privacy during consultations. Translation services were often required during consultations, as majority of the respondents were Iraqi and Afghan speaking. There were some drug stock-outs, however nurses often had painkillers and flu- medication and gave this to patients. No specific protocol for addressing GBV, case was discussed in team meetings, transfers done if required, and the survivor was often referred to a psychologist. Living rooms, consisted of bunks of 4–6 people, with males and females and mixed groups of people. No case of GBV consultation observed during duration of ethnographic work. However, there were reported cases handled earlier prior to commencement of observation
Centre 2	Located in the centre of the city in Gent, Capacity of 85 Majority of the population, unaccompanied minors of |Afghan origin. Mostly single males, no family present during the duration of observation	Open-access centre	Consultations with social workers and education workers, engaged in social activities with the refugees, cooking and outdoor activities, organized a sexual health workshop/ focus group discussion at the centre	9 months: May 2017 to March 2018, 5–8 h per week	An external mode of delivery for health care services, as medical services are not located in the centre. Most of the respondents had received positive responses for their asylum procedure and some were also classified as ‘medical cases’ with chronic diseases like Diabetes, Chronic Kidney failure or AIDS. Referrals to clinics was done and to GPs, as no medical service was available ‘in-house’. Social assistants were easily accessible, as their offices were located within the centre,
Centre 3	Located in Brussels, has a capacity of 850 people, population is mixed and has people from Asia (including the middle east) sub-Saharan Africa, Latin America, and Eastern Europe	The centre has gates, and a badge is used to enter and leave the centre, refugees had to take permission to leave the centre and were allowed to stay outside the centre for only a certain number of days. In many ways felt like a closed gated camp	Consultations with health care providers, multi-disciplinary team meetings. Informal discussions with refugees at the waiting room and the courtyard	1 year: May 2017 to May 2018: 6–10 h/ week	Medical centre available within the refugee centre, refugees who want to use the medical service are expected to come to the centre and book consultations between 10 and 12. ‘Less serious cases ‘are seen by the nurses, which means things like colds, cuts and bruises that need to be sutured etc., and more serious conditions that require treatment with prescriptions or a more thorough medical assessments are given dates for consultation with the doctor.

**Table 2:** Key characteristics of the centre

Type of centre	Service providers available ‘in house’	Availability of protocol to address violence	GBV / Torture referral pathway	Commonly used GBV / Torture interventions	Key challenges
**Centre 1**	Yes	No	Reports to doctor/ Social assistant, reviewed and then referred to psychologist	Referral to psychologist, transfer to another centre, to separate the perpetrator from the survivor in cases of domestic violence/ interpersonal violence	No defined protocol for addressing gender-based violence or torture. Clear pathways and action plans not defined. Made harmonization of practices and responses difficult across different service providers
**Centre 2**	No	No	Discuss with the social assistant and then refer to a psychologist if needed/requested	Referral to specialists, transfer survivor to a quieter centre, if survivor has symptoms of PTSD or other mental health problems	Disclosure was difficult and rare, especially as this centre had mostly males, stigma around gender-based violence and PTSD in males, made help seeking behaviour rare. No defined protocol and pathway of care was available
**Centre 3**	Yes	Yes	Refer to the doctor for physical bruises and then to the psychologist or an external organization for psychosocial support	Refer to psychologist, discuss experience of violence in a multidisciplinary team, transfer the survivor to another centre in the cases of domestic violence	Rates of disclosure was very low. Language translations served as a barrier as well during consultation, though efforts were made to employ translators and use on-line translation services. Consultation hours were specific and few service providers, and not all patients could see a doctor when needed Before the end of the observations, they had employed a psychologist ‘in-house’ that saw survivors of violence during consultation hours. It was hoped this would improve access to psychosocial counselling.

**Table 3:** Information about research participants

Type of respondent	Number
Social worker	4
Medical doctors and psychologist (1)	5
Nurses	3
Asylum seekers	13
Medical directors	2
**Total**	27

We stopped interviewing more research participants when thematic saturation was reached. Studies with a similar focus, have found thematic saturation to be reached at 12 participants [29]. In this paper, thematic saturation refers to a point, where analysis of new interviews or data reveal no new findings or insights that differ from that of prior interviews [30]. Research participants approached for an interview, were encouraged to take their time to review the informed consent form or think about the research, before agreeing to sign the informed consent form and be interviewed. For the asylum seekers recruited at the clinics, the research project was always introduced by a service provider, after the consultation was finished. They were assured that participation in the research project was voluntary and would not influence their asylum procedure or access to services. Information about the research was also made available in Arabic, French, Dutch, Farsi, Pashto, and Dari and placed in common areas in the different asylum centres. Translation of the research information and back translation was done with an Afghan refugee, who had practised as a medical doctor and was now working with refugees as a medical translator, he participated in some of the interviews and discussions of findings. A Syrian refugee was also employed as a research assistant to ensure there was reflection and reflexivity on some of the findings and we were able to incorporate the viewpoints of refugees and asylum seekers in the interpretations of the qualitative research. An additional Focus Group Discussion was done based on the request for a sexual health workshop by one of the centres. The workshop was done in English, Dari, and Pashto with five male asylum seekers at the centre, who were also interviewed individually. It was co-facilitated by an Afghan refugee who was a medical doctor and worked at the University as a medical translator. See Additional file 1, which provides details of the interview questions used.

For the analysis, we used an abduction approach, which focuses on finding explanations from observed facts’, using a combination of inductive and deductive methods [31, 32] qualitative content analysis [33] was done based on pre-identified codes based on the research questions developed by the researchers. For the data analysis, we used Atlas.ti, a qualitative research software that allowed us to classify our themes into code families and codes (categories and subcategories). This ensured that both researchers (EO and AJ) were able to code using the same frame of reference, discussions about codes and their meanings were discussed between the researchers [34]. We developed the theoretical framework and research hypothesis from a literature review and discussion with experts, we then proceeded to develop codes and code families to reflect the main concepts behind the theoretical framework. However, during the coding process, we recognized that our codes and code families were not necessarily sufficient to capture all the differing concepts. In those cases, we did open coding and then subsequently categorized the codes into code families that already existed or created new code families. Triangulation of qualitative data generated from the different qualitative research methods, information from the interviews and findings from observations done at these centres over a period of a year, was used to understand the pathways and ways social network members influence decision to access sexual and gender based violence services for SGBV survivors at the asylum centre [35].

## Results

We have described the key findings based on the theoretical framework described above.

### Survivor’s and network members’ attributes (factor a)

We classified several factors related to respondents’ family situation, their experiences of sexual and gender-based violence before and during their migration to Belgium, as well as, the structure of the asylum centre, including service provision and health system factors, as contextual factors. Twenty-seven respondents were interviewed during the research projects. All respondents were above the age of eighteen and were able to give informed consent. The service providers consisted of social workers, education workers (focused on life skills and supervising daily activities in the centre), medical doctors, nurses, and psychologists. The asylum seekers were from East and West Africa and West Asia. A third of the asylum seekers had at least a bachelor’s level of education and cited political unrest and economic reasons as some of their reasons for migration. Specific details about the countries they come from have been excluded to protect their anonymity and prevent stigmatization that might arise from conclusions of the study. During the interviews, issues around integration were identified as important by all the respondents but more by the social workers. This was defined as being more than just understanding the local language but also behaving in what was considered a ‘culturally acceptable manner’. The term ‘culturally acceptable’ was described in terms of adhering to an acceptable dressing style, manner of speaking, ‘ways of conducting oneself’ and hygiene. However, one could argue that these integration issues were not cultural per se, but more related to ideas around propriety in Belgium and conflicts with different behavioural attitudes and diversity.

‘ … *social network with local people. It is not an easy task. Lots of cultural differences, first they have a cultural shock, a lot of differences, … , you need too much time to integrate.*’ (Medical translator and doctor, Refugee)

*‘ … you walk on the street and you see, a lot of people say. We see these people (refugees) and we are afraid … that’s a small thing, I think the way you dress is less important than the way you conduct yourself.’* (Social worker)

The social worker discussed the underlying tension or fear among the local population, as what could be referred to as ‘the fear of difference’, specific issues raised were the ‘loud manner’ of some migrants, which were perceived as aggressive, or the unruly behaviour of migrant children from specific backgrounds.

The duration of stay in Belgium varied from 3 months to 7 years among the research participants, who were asylum seekers. However, there was no reported association between length of stay in Belgium and perceived level of integration. It is important to point out the complexities of integration in Belgium, which are heavily influenced by linguistic and regional politics and can be ether broadly defined as assimilationist or multi-culturist. The multi-culturist-interventionist type of policy and approach, which is more common in the Flemish region is characterized by compulsory civic and language classes, and a focus on migrants adhering to the Flemish identity. The assimilationist colour blind approach is more common in the Walloon region and has a policy that allows room for diversity, hence the lack of compulsory language classes. These differences may add layers of complexity to the definition of cultural integration in Belgium.

Most of the asylum seekers and health workers interviewed for this study were reflective while describing their experiences providing or accessing healthcare at the centres. Most of the refugees expressed an appreciation for the services they were provided at the different centres, while also expressing dissatisfaction at barriers, which are described later in this paper. The service providers also seemed to understand the budgetary and human resource challenges encountered in their provision of services and described different strategies for dealing with this.

#### Prior and current experiences of sexual and gender-based violence

Physical violence was the most reported type of sexual and gender-based violence among the respondents. Most of the respondents discussed this situation with the social workers. One of the most common barriers discussed was the cultural expectation or shame linked with sexual and gender-based violence (physical or sexual). Responses to reports of physical violence were varied, from ‘no action taken’ to provision of psychological counselling.

*Physical mostly, physical violence is often [reported by] men and women, but sexual violence either people are not disclosing, (or there are) rare cases, I have seen myself. I think this exists but might be because of shame, taboo, or cultural differences they are not going to disclose it.* (Research assistant, Male)

Some of the service providers reported instances of torture among the male refugees. They also discussed the difficulties with getting these men to share their experiences of trauma. While all the service providers recognized the importance of providing counselling and psychological care to the asylum seekers, most centres had no in-house psychologists present. External referrals were often required, as some of the asylum seekers had symptoms of post-traumatic stress disorder (PTSD).

*Yes, of course I would never say, this one has been tortured, but I could say to the reception or to the nurses, don’t disturb us now, because it is a heavy conversation. So that would create like a kind of bubble … Especially because we had men who had been raped, they would never talk about this in their interview, because of the shame and trauma, it was too big. And in this way, a psychologist … we had a conversation about torture, and I would never stop it at that, because then the story is out, but the evil spirit is also out. So, we need to provide counselling and afterwards … There were some particularly good psychologists, (with) whom I would make sure that the people would go there for follow up and for treatment.* (Medical doctor, Female)

From conversations with service providers, it seemed that men were more likely to report their experiences of sexual and gender-based violence as torture and women as experiences of sexual and gender-based violence.

Sexual violence: Though there were instances of sexual violence, disclosure was difficult and often dependent on the attitude of the health professional. Health care professionals who probed deeper for sexual violence risk, were more likely to have patients disclose their experiences.

… I see a lot, and I think not all are … I try to ask, it is not easy to ask directly, for women it’s not easy to answer … I am clear that lot of people (have experienced) sexual violence … not only women, we have a lot of young men from Afghanistan (have experienced) sexual violence too. So, I try to ask *whenever it’s possible. But I see a lot, more than normal with my consultations...* (Medical doctor, Female)

The medical doctor quoted above repeatedly stressed on the sensitive nature of screening for SGBV, a strategy she used, was to ask a lot of general questions before discussing sexual violence. Another medical doctor affirmed:

*… A lot. Oh, they were very open about it. But never, almost never from the beginning, of course. That's why I think this intake was ok, because we wanted to give them the feeling that there was an opportunity to talk about it. And for many times we opened, we were very active in starting a conversation about this* (Medical doctor, Female).

This comment reaffirms the importance of screening for experiences of sexual and gender-based violence, as this provides an ‘opening’ for asylum seekers to discuss their experiences.

#### Common health problems: psychosomatic symptoms

Some of the most common health problems, the respondents presented with at the health clinic were psychosomatic, we use the term ‘psychosomatic’ to refer to physical conditions and symptoms that are an expression of the emotional or psychological state of an individual’. For example persistent body aches, with no other underlying causes or explanation apart from repeated psychological trauma or stress [36], it was rare for them to present at the clinic and directly report their experiences of SGBV They only agreed to share their experiences of violence after several discussions. Psychosomatic symptoms were often related to experiences of trauma, during their migration journey or in their home country, and symptoms of post-traumatic stress disorder.

… *it is mostly combination of anxiety related problems including sometimes Post Traumatic Stress Disorder, and sometimes more severe problems like psychosis. Sometimes stomach pain, breast pain, and anxiety related problems like flashbacks, and depression of course. Often related to a combination of traumatic experiences and losses, born in uncertain situations, difficult events..*. (Psychologist, female)

*… long time, long time. But support here (in Belgium) because sometimes I couldn’t sleep, I would dream about it, but they gave me some tablets to help me sleep. I used to take them but after some time I stopped because I wanted to sleep in a natural way. I am afraid, still have them with me. Sometimes it happens, I can spend one week without sleep, morning, evening I don’t sleep, and I am strong. But that is not life...* (Asylum seeker, SGBV survivor, female)

These findings show that service providers need to spend sufficient time discussing with their patients/ clients and probing for experiences of violence, as discussed above. Survivors of SGBV, might be more vulnerable to repeat experiences of SGBV, especially as they are far from home and live-in asylum centres. It also requires that most medical centres ensure that they have the right referral pathways, so they can ensure survivors of violence identified have the right access to psychosocial support. Survivors of SGBV, might be more vulnerable to repeat experiences of SGBV, especially as they are far from home and live-in asylum centres.

### Co-factor a: contextual factors

Contextual factors such as cultural norms and health system factors were also reported by most interviewees as influential in their decision making to access healthcare services.

#### Family support and cultural norms around SGBV

Family members of a survivor of violence could influence their coping strategies and attitudes towards reporting their experience of violence. In a case of a female survivor of sexual and gender-based violence, her mother’s support was highly valued as it gave her the psychological support required. Her mother told her to cope with the experience of domestic violence, as it was a cultural norm for husbands to sometimes beat their wives. This example outlines the complexity of family relationships and reporting patterns. Family members might be able to provide functional support to survivors of violence and aid them in coping with stress and psychological effects, and still discourage them from reporting. In some cases, the family member might be the main aggressor, and more interested in preventing the reporting of violence. Hence, network interventions that focus on key players (very influential network members) would have to take into account the complexities that exist in social networks with family members.

#### Asylum application process

The asylum application and process arose from most of the interviews as a crucial component and an indicator of the well-being of asylum seekers. Most of the respondents (asylum seekers and service providers) spoke of the difficulties of being ‘in transition’ being moved from one centre to another, while awaiting the decision on their asylum. This was a factor that had a significant effect on their psychological well-being. These changes and frequent movement among people still within the asylum process, might make it difficult for sexual and gender-based violence survivors to access needed care, as well as needed follow up, psychosocial counselling and medico-legal procedures.

*‘there’s a huge difference, in terms of the challenges, the uncertainty, you often see it in the chain from the asylum procedure, to obtain a status, that makes a huge difference both in the positive and the negative sense, it impacts your mind … positive is what gives security and safety, the feeling that I can now start my life, Negative (asylum status) is that you begin to lose many support structures in terms of the asylum centre and that feeling of stability … ’*(Service provider, psychologist, female).

#### Availability of health service providers and treatment

In general, health service providers, social workers, nurses, and doctors who provided services to SGBV survivors referred the survivors to psychologists, with their consent. Among the three centres that the observations and interviews were carried out, none had ‘in house’ specialized treatment and forensic services for SGBV services, so all survivors had to be referred to external services. We have classified health services as part of the concept of ‘context’ because they are sometimes the only formal source of support and help, which SGBV survivors can access. The health service providers had specific opening times, and these were sometimes identified as a barrier to accessing health care.

There was a reported disconnect between refugees and service providers’ expectations about availability and opening hours. The working hours of the centres were sometimes perceived as a barrier for access to healthcare for most of the asylum seekers. However, for the service providers it was especially important for them to have that structure to enable them function effectively. When the opening times were not respected, this was often viewed as ‘crossing boundaries’ or ‘being disrespectful’.

*… But I always give the signal that it is possible to come, if they want something, and I keep reminding them. But they also have to follow the rules, because it is not because you are a loner and one time you make a decision you come and ask for help, if you do it in the break, it is break time, you are not getting special treatment …* (Social worker, female)

*… yeah, yeah, it’s been easy (to access healthcare). The problem is that they just open for two hours. But the service is good when they try to do everything, and when they can’t they transfer you to the big hospital. If you don’t have an appointment, they can’t. But they do their best’* (Asylum seeker, female)

It is important to note that the concept of availability is not the same for service providers and for asylum seekers (service users), and this could influence how survivors of violence perceive the availability of support for them. It is important that service providers take into account that specific vulnerable groups, for example, survivors of violence might require access to support services that extend beyond daily working hours. Creating alternative services, like chat lines or emergency support could make a difference in access to healthcare for these groups and mitigate harmful consequences. Also, from the human resource perspective, understanding the need for extra hours and more staff, referral pathways and adequate compensation for staff, can prevent burn out and motivate service providers.

Some of the respondents reported difficulties in accessing services due to the lack of sufficient human resource. This problem also limited the ability of service providers to provide sufficient assistance and support to the survivors of violence

*… I know a lot of people came once or twice to the nurses and then say I don’t want to come back because it not good. Lots of people think that medical services (are) not good because it’s difficult to access the doctors because there are lot of people, we don’t have sufficient spaces and workers … (*Medical doctor, female)

#### Lack of trust and ambivalence from service providers

Trust arose as an important factor, that could also be enabling and encourage disclosure of experiences of violence. It was also a barrier when there is a lack of trust present.

*… Many are willing to discuss but there are parts that are hard to express. And it largely depends on the situation they were in. if they are at peace to talk in a quiet stable situation. For some it’s hard to talk about because it reveals lots of emotions. Sometimes they feel like avoiding those emotions because they are too tough to feel. It depends. I feel there is lot of distrust preventing them to talk about it. Protecting themselves. It depends* … (Psychologist, female)

In some cases, when the survivors of violence reported their experience of violence, they were met with ambivalence from the service providers which discouraged reporting of violence.

*‘[breathes] last time I was passing by the block F, I had come to see my assistant, I heard in one office one lady telling that one guy was abusing her. But sometimes these things happen. [And even if they talk to the assistant, they do nothing]. I don’t know … they just give them advices … like I heard even before I came to PC [centre] one lady was telling that one guy was abusing her, the assistant was laughing, did nothing [reports of abuse taken very lightly, no redress l [sic] … but I heard that later they changed her room.* (Asylum centre, Male)

Medical centres that provide care to asylum seekers or undocumented migrants, often have a huge demand with limited resources, making it difficult for many asylum seekers and undocumented migrants to develop relationships of trust with their service providers.

However, it is possible for these relationships to be built over time. For example, most asylum seekers interviewed developed good relationships with their social workers because of sustained interactions over time. In centres, where asylum seekers saw a particular health provider over a long period of time, there was also more trust between the provider and asylum seeker.

### Factor and co-factor B: Network’s member reaction to reporting and effort required by the network members to support or oppose

From the interviews, efforts required by family members, friends, and service providers, did not come up as an issue or factor that influenced the level of support. However, in one interview with an asylum seeker, she mentioned that it was impossible to get support from her father because contact (via phone) would put him at risk. In this case, the effort and personal cost required by her father to provide emotional or financial support to her, was too high. The same fear of persecution of family members, arose from other conversations with political asylum seekers who had survived other forms of trauma and whose families were still living in their home country. In most of these cases, there was no contact from family members.

From interviews with service providers, the personal cost of working extra hours or providing care during ‘lunch hours could serve as a barrier to access. Some of them were unwilling to do this. Hence, our earlier recommendation for training, recognition, and compensation of extra working hours for service providers.

### Factor C: costs of effort for the survivor to report or not

‘*Yeah. And also, if the, for example, there is abuse in one family, ehm, and the family is here, there is more pressure from the family members not to tell anything instead of, there is woman, or a man, coming here and has been abused, but here she is alone, maybe there is less pressure from the family’* (Social Assistant, Female)

In many situations, especially if the asylum claim is made by a whole family, and there is an incident of SGBV, where the perpetrator is a family member, it would be very difficult for the survivor to disclose and seek help for the incident because of the implications (the asylum process) for the whole family. In other instances, the dependence many asylum seekers have on family and social networks for emotional and financial assistance, can also negatively influence their ability to disclose incidents of SGBV within the family circle. Understanding these dynamics can be helpful for service providers and researchers, in understanding the barriers to access to healthcare.

Family members of a survivor of violence could influence their coping strategies and attitudes towards reporting their experience of violence. In a case of a female survivor of domestic violence, her mother’s support was highly valued as it gave her the psychological support required. Her mother was very influential within her network, but her mother discouraged her from reporting and seeking help for her experience of SGBV from formal sources. Her mother told her to cope with the experience of violence, as it was a cultural norm. This example outlines the complexity of family relationships and reporting patterns. Family members might be able to provide support to survivors of violence, which is helpful with coping with stress and psychological effects, and still discourage them from reporting. In some cases, the family member might be the main aggressor or an important influential member of the family. Hence, the cost of the survivor reporting the incident, would have a negative impact on the family relations, as well as a personal cost to the survivor and family member.

### Co-factor C: degree and centrality measure of network members

Among asylum seekers interviewed, it was difficult to assess which asylum seekers were ‘central’ to their network and were key players. However, it was clear from some interviews, that certain people had more authority and more contacts with different asylum seekers than others. Also, during collection of pilot data and ethnographic work, it was clear that some family members’ or friends’ opinions were more valued than others in decision making. In some instances, especially when it was about navigating the legal and social system in Belgium. The perspective of the social assistant was more valued, or another asylum seeker with more years of experience of living in Belgium. The implication this has for SGBV survivors, is that if SGBV interventions are directed to individuals, and influential members of their network oppose their decision to access health care, this will make it very difficult for the asylum seeker tor receive the necessary emotional and physical support required and might even result in isolation from other members of their network.

#### The bridge

During the interviews, we identified people whom Valente (2012) refers to as people with bridging properties within a network, they were often people who had been in the centres for less than 6 months, had few social network members, were bi-lingual and had friendship networks that were heterophilic. In a microsystem, were most people sought friendships with people they viewed as being similar, bridges were often people who had friendships across ‘cliques. Though they had close friendships with people from their own country, they were likely to identify people from different countries as being part of their friendship network. They would not be identified as key players/ opinion leaders in a network analysis, but during my interviews and subsequent informal conversations with them, they were the ones who expressed more of an interest in our research project and proffered specific recommendations to address gender-based violence and its effects, that were based on peer-support. I ended up engaging some of these people as volunteers in my research project. I think these are the people with the greatest potential to effect change, especially in complex network structures, like those found in the centres we worked in.

‘*No, no [responds to question about having friends from the same country] … . from other places like Rwanda, Congo, Niger, Ivory Coast, Kenya, Morocco, yeah, [the friendships are helpful]...One day, was talking to a staff at PC [centre] regarding as we are coming from different countries, we have different cultures, many things, I was telling them like to do one meeting for all people at PC … for some people who, how to say it, who in their life had difficult times, they want to be consulted [and speak about it].. because talking about it, you will feel well …* (Male, asylum seeker)

The asylum seeker we spoke with above supported the idea of getting asylum seekers of different nationalities together and consulting with them to jointly develop solutions to address their past experiences of violence and trauma and develop sustainable solutions.

##### Limitations of the study

This study focused on the experiences of asylum seekers who had formally lodged an application to the Belgian government and hence had access to social services pending the result of their application, and in so doing we were unable to document the experiences of many undocumented migrant that also have experiences of SGBV. In most of the centres there was a mix of asylum seekers from Asia, sub-Saharan Africa, and Latin America. Difficulties in ensuring that we did not generalise some of their stories because of the differences in culture and migration experiences were encountered. We discussed some of the initial findings with two asylum seekers who were part of the analysis and data collection, and had lived in one of the centres, to ensure that we were reflexive about our interpretations.

## Discussion

Network –oriented interventions have been widely used in public health for different types of interventions including but not limited to smoking cessation, cervical cancer screening, diet and weight management and HIV prevention with different target populations, for example, sex workers and intravenous drug users [37]. However, there is limited evidence of the use of network interventions among vulnerable groups like asylum seekers and refugees. The use of network theory or social network-based interventions involving asylum seekers and refugees requires an understanding of the context and the different factors within network interactions, which might influence decisions to access health care. This aligns with other findings from a study done by UNHCR on intimate partner violence interventions, which found that implementing a peer based intervention without effective community engagement and understanding of the context, would negatively affect the success of the intervention [38]. Our research findings indicate that the quality and perceived importance of connections is a key factor to developing peer based or network interventions, as compared to the number of connections. The popular assumption that, the presence of friends and family confers a protective barrier to sexual and gender-based violence is not always correct as provided by examples from our interviews, and this kind of information can be teased out from respondents during the screening process. This finding is similar to Llyod’s work on refugees in Australia, which shows that family and friends can be important sources of information and support, but specific harmful cultural beliefs shared among close knit or cohesive networks can be a deterrent to accessing health care [39] . Although, in some cases where family and friends are supportive, this might provide the survivor of violence with enough impetus to report their experience and seek health care.

Context, specifically the existing asylum policies and processes, health system barriers as well as other barriers like language are of equal importance in developing network-based interventions. Peer support might be useful in providing information, emotional support, and resources but challenges experienced during the asylum process, could have a deterring effect on the willingness to access health care. The psychological distress experienced by many refugees during their migration process, as well as the uncertainty regarding their status in the new country can take its toll on their psyche, and ability to utilize existing Services [40] The language barrier is an often overlooked factor but is significant in ensuring access to health care, information and resources [41]. In some settings, translators where used when available, as well as inter-cultural communicators, but these interventions are not systemic or widely used in all asylum centres [42].

Expanding the definition of networks and incorporating service providers, like social workers, advocates, doctors and nurses into developing support interventions is important as discussed earlier in the paper, and identified in other studies done with refugees in high income countries [43]. For some refugees, these contacts serve as the first and only source of information and resources. Also, addressing values and assumptions about refugees among social workers and doctors is important. This is an overlooked step in many asylum centres, as refugees might have specific challenges, which are different from the general population, which social workers and doctors in the host country might be unfamiliar with. Understanding concepts like resilience and the need for better screening processes especially during the intake process at asylum centres might improve identification and adequate referral processes for survivors of different forms of violence at the centres, especially for mental health issues like depression and post- traumatic stress disorder arising from experiences of SGBV [44].

## Conclusion

This study describes some of the network factors that influence the decision to seek formal care by asylum seekers who are SGBV survivors. Our findings draw attention to the importance and role of peer support, in access to health care, and the importance of understanding the nature of the social network of asylum seekers, before implementing a peer support or peer-led program. The effectiveness and applicability of such interventions is heavily influenced by the context: existing asylum policies, availability of health services and the ‘centrality’ of their close social contacts, among other factors.

Through the interviews with asylum seekers and service providers, we identified pathways, through which social network members influence the decision making of SGBV survivors. This has implications for community and peer based interventions, as it is not sufficient to work with peers, without effective community engagement to understand the context of the target population [38]. For example, understanding the personal and societal cost reporting would have not just for the survivor, but for the close network members that would support the survivor’s decision to report, will also have implications on the survivor’s decision to seek help and utilize existing peer-based interventions.

The context of the survivor which includes the availability of supportive asylum related health policies, SGBV care services for the survivor at the asylum reception centres or through referral, is also an important factor, as well as the availability of trained counsellors, and staff that have the skill sets, time and ability to screen for SGBV survivors. This will have an impact on disclosure rates and also utilization of SGBV care services [6].

Our model proposed earlier, can provide a way of mapping these different factors and evaluating the different ways a peer-based intervention can address these factors to ensure that the survivor of SGBV is able to overcome challenges to reporting their experience of SGBV and seeking the required help. Especially for asylum seekers, who may likely not have as much social support in their host country, understanding these different factors would help in developing more responsive and effective programming to address their needs for care, especially for SGBV survivors.

## Supplementary Information

**Additional file 1.** Interview guide for asylum seekers and service providers. Interview guide used in interviews of asylum seekers and service providers.

## Data Availability

The datasets generated and/ analysed during the current study are not publicly available, as the data for this study are transcript interviews with asylum seekers (a vulnerable group) and service providers with pseudonymized information that might be re-identifiable. However, they are available from the corresponding author on reasonable request.

## Abbreviations

FEDASIL: Federal Agency for the Reception of Asylum seekers
PTSD: Post-traumatic stress disorder
SGBV: Sexual and gender-based violence
IPV: Intimate Partner violence
