# Supplementary material for: The potential role of network-oriented interventions for survivors of sexual and gender-based violence among asylum seekers in Belgium
Source: BMC Public Health. 2021 Jan 5;21:25. doi: 10.1186/s12889-020-10049-0 (PMC7786941; doi:10.1186/s12889-020-10049-0)
Supplement: Supplementary file 1 — Additional file 1. Interview guide for asylum seekers and service providers. Interview guide used in interviews of asylum seekers and service providers. [file 12889_2020_10049_MOESM1_ESM.docx]

**APPENDIX 1: Information sheet and In-depth interview guide for Asylum seekers**

**Title of the study: Assessment of conditional preferences and decision making in utilization of SRH services among SGBV survivors: Developing predictive models using game theory, a study among asylum seekers in Belgium**

**IN-DEPTH INTERVIEW GUIDES ASYLUM SEEKERS/ SURVIVORS**

1. **Background and community**

| **Interview questions and prompts** | **Comments** |
| --- | --- |
| - 1. Please introduce/ tell me about yourself (age, sex, marital status, present living conditions and socioeconomic status)? | PROBE to understand characteristics of social network and experiences of migration (reference sociodemographic questionnaire at the last page)  PROBE to understand experiences of accessing services at the different centres and extent of social support received to access services |
| - 1. Please describe the experiences that led you to seek asylum or refugee status in this country? |  |
| - 1. Please describe your experiences since you arrived in this country (focus on challenges and opportunities)? |  |
| - 1. Do you currently live alone or have friends or family here. Please describe your existing relationships with friends and family here? |  |
| - 1. Do you feel like you are part of a community or social support system here? Please explain the reasons for your answer. |  |

1. **Experiences of accessing services at the asylum/ refugee centre**

| - 1. How did you find out about this asylum or refugee centre? | PROBE to understand health seeking behaviour and information sources.  PROBE to understand some of the challenges and enabling factors that influence seeking services |
| --- | --- |
| - 1. Please describe the experiences that led you to seek services at the asylum/ refugee centre? |  |
| - 1. What kind of services have you accessed at the centre? |  |
| - 1. Have you accessed services at other centres, please describe? |  |
| - 1. How easy or difficult was it to access these services? |  |

1. **Type of support received after experience of violence (Skip to question 5, if not a survivor)**

| **Interview questions and prompts** | **Comments** |
| --- | --- |
| - 1. We know you had an experience of violence, which we know you may or may not want to talk about. This is entirely up to you but we would appreciate it if you share the effects that the experience has had on your life? | PROBE to understand sources of information and relative importance of the source of information within the social network of the survivor.  PROBE to see if the survivor sought any kind of help? Even if it was not institutionalised (i.e. Support group, religious or talked to close friend or family)  PROBE gently to see if experience of violence led to complications: sexual and reproductive health or mental health complications.  PROBE to understand the influence of other factors (use the Andersen’s model of health behaviour as a guide.) |
| - 1. Did you know the person who did this to you? |  |
| - 1. If so, please could you describe your relationship or how you met the person who did this to you? |  |
| - 1. Did you try to find services after this happened to you? |  |
| - 1. Did you feel you needed someone to help you with this? |  |
| - 1. Did you access services after it happened, if so, please describe what services? |  |
| - 1. If you accessed services, what were your experiences of receiving these services? |  |
| - 1. Are there other factors that influenced your decision to seek help or assistance? If so, please discuss |  |

1. **Social network and support characteristics**

| **Interview questions and prompts** | **Comments** |
| --- | --- |
| - 1. Did you feel a need to speak with anyone, after your experience? | PROBE to understand type of support received and health seeking behaviour  PROBE to understand type of support received and disapproval, if applicable  PROBE to understand scope of stakeholders and members of social network of refugees/ asylum seekers and their social networks. |
| - 1. Did you speak with anyone, among your group of family and friends after it happened? |  |
| - 1. Whom did you tell and why? |  |
| - 1. Did discussing your experience with these persons change your relationship with them? If so, please describe how? |  |
| - 1. What kind of advice did you get from the people you told? |  |
| - 1. Among the people you told about the experience, whose advice mattered the most to you? |  |
| - 1. Did you feel you received support after it happened? If so, please describe the kind of support and the relationships you had with the people who provided support |  |
| - 1. Did anyone not support you as you had wished/ express disapproval? If so, please describe your relationship with this person and their expressed reasons of disapproval |  |
| - 1. Do you think the support or disapproval you received would have been different, if the circumstances of your experience were different? If so, please describe? |  |
| - 1. Did any of the advice/ support you receive weigh in, on your decision to seek help or not? If so, how? |  |
| - 1. Are you currently still accessing support or services? |  |
| - 1. Did you seek support/ information from other sources? Please describe |  |
| - 1. Please describe how you currently feel or are coping with what happened? |  |

1. **Knowledge of other sexual violence survivors and relevant interventions**

| **Interview questions and prompts** | **Comments** |
| --- | --- |
| - 1. Do you know of any other people in your community, who also experienced the type of violence you did? | PROBE to assess if there are existing community based interventions with an underlying community support strategy.  PROBE to understand underlying assumptions beneath these interventions and the understanding of the influence of social networks |
| - 1. If so, please describe your relationship with them and how you heard about their experience? |  |
| - 1. Do you know if these people received support from their group of friends/ families or other stakeholders? Please describe their experiences |  |
| - 1. Do you know if these people received disapproval from their group of friends/ families or other stakeholders? Please describe their experiences |  |
| - 1. Do you feel the support or disapproval these people experienced, influenced their decision to seek or not seek help, after their experience? Please describe how? |  |
| - 1. Do you think other factors also influenced this? Please describe which ones and how? |  |
| - 1. Do you know of any support interventions for gender based violence survivors in your community? Please describe them? |  |
| - 1. Have you used them or anyone you know? Please describe your experiences and if they were helpful or not? |  |
| - 1. Do you have any recommendations for how supports services for gender based violence survivors can be organised or better developed? |  |

1. **Verification of stakeholder maps**

Present the stakeholder maps that were developed during the focus group discussions with the refugees, and ask the gender based violence survivors to discuss the different actors and rank/ adapt based on their own experiences, and the influence they had on their ability to cope after their experience of violence.

**Background: Socio-demographic characteristics**

*In this section, we ask about some background characteristics and information about you.*

1. How old were you on your last birthday?

Please indicate: -------------------------------------

1. What is your gender?

Please indicate: -------------------------------------

1. What is your nationality?

Please indicate: ------------------------------------

1. What is your Ethnicity?

Please indicate: -------------------------------------

1. Please indicate your marital status

Single Divorced

Engaged Widow

Living together Widower

Married Other

Separated

1. What *level* of education did you achieve? Please indicate.

None Others:---------

Primary

Secondary (low)

Secondary(high)

Higher Education (Vocational)

Higher Education (University)

1. What did you do as your daily activity in your country (country of origin)?

Student H ll time)n jo University)gin)n -----------------
ouse wife/man

On the job market Volunteer

Paid work, part time Unable to work

Paid work, full time Specify why:

Self-employed Retired

Other

1. What daily activities do you do in this country (host country)

Student H ll time)n jo University)gin)n -----------------
ouse wife/man

On the job market Volunteer

Paid work, part time Unable to work

Paid work, full time Specify why:

Self-employed Retired

Other

1. Years of working: ________
2. Years of living in this country: _________
3. Years as an undocumented migrant/asylum seeker/ refugee: _______________
4. Specify what kind of accommodation you have: ------------------------------
5. How many people live with you presently? ___________
6. Please indicate your income level (monthly)?
7. Do you have access to sexual and reproductive health services in your community?

Yes (continue)

No

1. Do you have Internet access at home?

Yes No

*________*

**IN-DEPTH INTERVIEW GUIDES SERVICE PROVIDER**

**INTRODUCTION**

Good morning. My name is ………………………….. …………….

Thank you for agreeing to take part in this interview.

I am pleased you have agreed to make out time for this interview. I am a researcher and PhD student at the International Centre for Reproductive Health (ICRH), University of Ghent, Belgium. During the interview, I would like to discuss your experiences of providing services to asylum seekers/ refugees at this asylum/ refugee centre

I would like to keep a record of this discussion so that I don’t have to take notes, if this is alright with you? I like to follow what is being said and then go back later to review what you said again so I can accurately convey your ideas and opinions. I will transcribe our conversations but your identity and other personal identifiers will be anonymised. The recorded audio files will be destroyed after the completion of this study and the transcribed data kept till 5 years after the completion of the study.

The interview will last between 45-60 minutes.

Any information you provide during the course of the interview will be kept confidential and used only for analysis by the research team

Was all the information I provided you with clear? Please let me know if you have any questions about the research?

| Name of Interviewer |  |
| --- | --- |
| Identification Code and function of the respondent |  |
| Date of Interview |  |
| Duration of the interview |  |

1. **Background of service providers and centres**

| **Interview questions and prompts** | **Comments** |
| --- | --- |
| - 1. Please introduce yourself, and describe your role within this organisation? | PROBE to find out length of service, experience and kind of services provided.  PROBE to assess level of engagement or experience with working with sexual violence survivors  PROBE to assess the socio-demographic characteristics of people who access services at the centre |
| - 1. Please describe the different types of services provided at your centre? |  |
| - 1. Please describe the services you are responsible for providing? |  |
| - 1. Please describe the client pathway or protocol for accessing services at your centre |  |
| - 1. What type of groups access services at your centre? |  |
| - 1. Please describe the sort of services that are sought commonly at your centre? |  |

| - 1. Do you often see gender based violence survivors at your centres? | PROBE to understand how services are accessed at the centre.  PROBE to understand service seeking behaviour of gender based violence survivors that access care at the centre. |
| --- | --- |
| - 1. If you do, how many of them are sexual violence survivors? Please describe their characteristics? |  |
| - 1. Is your centre usually the first point of call or are they referred from somewhere else? Please explain your answer |  |
| - 1. What services are mostly sought out, by the gender based violence survivors that come to your centre? |  |
| - 1. What is your centre’s protocol for providing services to gender based violence survivors? |  |
| - 1. Do you refer gender based violence survivors that come to your centres to other services? |  |
| - 1. If you do, what are the referral and evaluation procedures? |  |
| - 1. Is it easier to refer asylum seekers, compared to refugees or undocumented migrants? If so, please describe the differences between the groups. |  |

1. **Care seeking behaviour and characteristics**
2. **Factors that influence access to health care**

| **Interview questions and prompts** | **Comments** |
| --- | --- |
| - 1. What factors do you think influence access to health services for refugees/ asylum seekers | PROBE to understand the different barriers and enablers to access (See Appendix for Andersons model on health care behaviour)  PROBE on differences of types of social network and level of support based on the age, gender and social circumstance of the survivor.  PROPOSE different scenarios, to assess context related differences. |
| - 1. Are these factors same or different for gender based survivors, also for sexual violence survivors? |  |
| - 1. Do you think these factors are different based on the gender, age and ethnic group of the survivor? Please explain the reason for your answer? |  |
| - 1. What are some of the mitigating strategies that can be used among survivors of gender based violence? |  |

1. **Social network and support characteristics**

| **Interview questions and prompts** | **Comments** |
| --- | --- |
| - 1. Do you think, based on your experience that social networks play a part on health decision making of refugees? | PROBE to understand scope of stakeholders and members of social network of refugees/ asylum seekers and their social networks. |
| - 1. If you do, please could you describe what you think the social networks are made up of? |  |
| - 1. Do you feel service providers are part of the social network? |  |
| - 1. If so, please could you describe your role within the social network? |  |
| - 1. Please describe the components of the immediate social networks of the sexual violence survivors and refugees/ asylum seekers that access services at your centre? |  |
| - 1. Who do you think are the most influential actors within the social network? |  |
| - 1. What do you think is their influence on decision making among sexual violence survivors? |  |
| - 1. Do the survivors accessing care report sources of support or discouragement from their social networks to access services? |  |
| - 1. Is the kind of support different based on their age, gender or sex, based on your experience? |  |
| - 1. Is this different when the perpetrator is known or unknown by the sexual violence survivor? |  |
| - 1. How many of them come accompanied? Please describe the relationship of the sexual violence survivor with the people who usually accompany them? |  |

1. **Experiences of working with interventions focused on refugees**

| **Interview questions and prompts** | **Comments** |
| --- | --- |
| - 1. Are there current interventions within your organisation that are focused on refugees/ asylum seekers that are sexual violence survivors? Please describe? | PROBE to assess if there are existing community based interventions with an underlying community support strategy.  PROBE to understand underlying assumptions beneath these interventions and the understanding of the influence of social networks |
| - 1. Are there current interventions among other organisations that you are familiar with, that are focused on refugees/ asylum seekers that are sexual violence survivors? Please describe? |  |
| - 1. Do social networks of the target population of your intervention play an important role in influencing these programmes? If so, describe |  |
| - 1. Do you have recommendations on ways, working with and within the social networks of sexual violence survivors can improve uptake of sexual and reproductive health services? |  |

1. **Verification of stakeholder maps**

Present the stakeholder maps that were developed during the focus group discussions with the refugees, and ask the health service providers to adapt and discuss the hierarchy of the social networks based on their experience, including other external actors, like donor or aid agencies.
